# Ten Rules for Conducting Retrospective Pharmacoepidemiological Analyses: Example COVID-19 Study

**DOI:** 10.3389/fphar.2021.700776

**Published:** 2021-07-28

**Authors:** Michael Powell, Allison Koenecke, James Brian Byrd, Akihiko Nishimura, Maximilian F. Konig, Ruoxuan Xiong, Sadiqa Mahmood, Vera Mucaj, Chetan Bettegowda, Liam Rose, Suzanne Tamang, Adam Sacarny, Brian Caffo, Susan Athey, Elizabeth A. Stuart, Joshua T. Vogelstein

**Affiliations:** ^1^ Department of Biomedical Engineering, Institute for Computational Medicine, The Johns Hopkins University, Baltimore, MD, United States; ^2^ Institute for Computational & Mathematical Engineering, Stanford University, Stanford, CA, United States; ^3^ Department of Internal Medicine, Division of Cardiovascular Medicine, University of Michigan Medical School, Ann Arbor, MI, United States; ^4^ Department of Biostatistics, Johns Hopkins Bloomberg School of Public Health at Johns Hopkins University, Baltimore, MD, United States; ^5^ Ludwig Center, Lustgarten Laboratory, Howard Hughes Medical Institute, The Johns Hopkins University School of Medicine, Baltimore, MD, United States; ^6^ Division of Rheumatology, Department of Medicine, The Johns Hopkins University School of Medicine, Baltimore, MD, United States; ^7^ Graduate School of Business, Stanford University, Stanford, CA, United States; ^8^ Health Catalyst Inc., Salt Lake City, UT, United States; ^9^ Datavant Inc., San Francisco, CA, United States; ^10^ Department of Neurosurgery, The Johns Hopkins University School of Medicine, Baltimore, MD, United States; ^11^ VA Health Economics Resource Center, Palo Alto VA, Menlo Park, CA, United States; ^12^ Department of Biomedical Data Science, Stanford University, Stanford, CA, United States; ^13^ Department of Health Policy and Management, Columbia University Mailman School of Public Health, New York, NY, United States; ^14^ Department of Mental Health, Johns Hopkins Bloomberg School of Public Health at Johns Hopkins University, Baltimore, MD, United States

**Keywords:** drug repurposing, retrospective analyses, observational study, COVID-19, pharmacoepidemiology

## Abstract

Since the beginning of the COVID-19 pandemic, pharmaceutical treatment hypotheses have abounded, each requiring careful evaluation. A randomized controlled trial generally provides the most credible evaluation of a treatment, but the efficiency and effectiveness of the trial depend on the existing evidence supporting the treatment. The researcher must therefore compile a body of evidence justifying the use of time and resources to further investigate a treatment hypothesis in a trial. An observational study can provide this evidence, but the lack of randomized exposure and the researcher’s inability to control treatment administration and data collection introduce significant challenges. A proper analysis of observational health care data thus requires contributions from experts in a diverse set of topics ranging from epidemiology and causal analysis to relevant medical specialties and data sources. Here we summarize these contributions as 10 rules that serve as an end-to-end introduction to retrospective pharmacoepidemiological analyses of observational health care data using a running example of a hypothetical COVID-19 study. A detailed supplement presents a practical how-to guide for following each rule. When carefully designed and properly executed, a retrospective pharmacoepidemiological analysis framed around these rules will inform the decisions of whether and how to investigate a treatment hypothesis in a randomized controlled trial. This work has important implications for any future pandemic by prescribing what we can and should do while the world waits for global vaccine distribution.

## Introduction

Imagine we are only halfway through 2020; the COVID-19 pandemic is raging, and widespread vaccination is thought to be at least a year away. Treatment ideas abound for COVID-19, and around the world more than 2,000 clinical treatment trials have been initiated to begin testing a wide variety of drugs hypothesized to help infected patients. Unfortunately, constrained resources can only fund some subset of the investigator-initiated trials; hence, trials resourced to begin patient enrollment must be chosen judiciously based on the soundness of the medical hypothesis, the availability of preclinical evidence, and the trial’s feasibility, cost, and potential impact. It is in this environment that you have arrived with a novel idea for an effective pharmaceutical intervention for COVID-19 (or the next pandemic).

The gold-standard way to evaluate your hypothesis is a randomized controlled trial (RCT), but that takes time and resources you (and the world) may not have at the moment. In fact, the window to pursue your trial is limited as interest (and resources) will increasingly focus on progress in vaccine development. Assuming your trial would be ethically permissible and otherwise feasible (e.g., reasonable follow-up periods and realistic recruiting goals), is there anything you can do right now to investigate your hypothesis and determine the priority of testing it in an RCT? There are three common types of retrospective studies to consider, each of which uses observational data: cross-sectional studies, case-control studies, and cohort studies. This paper provides a framework for investigating your pharmaceutical hypothesis carefully and responsibly using a retrospective cohort study. Beyond just advocating for a clinical trial, your investigation can inform many of the decisions regarding the details of a clinical trial (e.g., which drugs and dosage levels to test), as well as who is most likely to benefit from your treatment; all of this may influence how stakeholders choose to prioritize your trial. A retrospective analysis focused on today’s disease (even after widespread vaccination) can also improve our understanding and preparedness for a novel disease we encounter in the future; completed studies targeting readily available treatment options in a related disease could help save countless lives when the next pandemic strikes and the world is again waiting for a vaccine.

**FIGURE 1:**
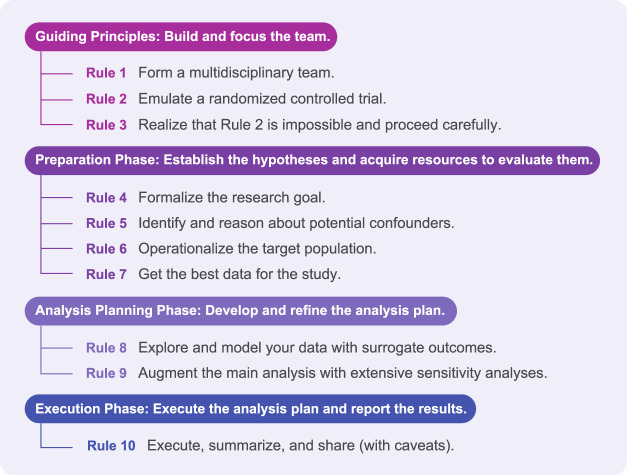
The first phase of the 10 rules involves building the right team to envision the perfect trial and then consider the limitations of an observational study. The study then enters a preparation phase in which the details of the study are specified: hypotheses, which population to target, essential confounders to observe, and which data sets might support the study criteria. In the analysis planning phase, the objective is to refine and validate the study definitions and selected methods without being influenced by real results. Finally, the study concludes when the study is run, carefully summarized, and reported accurately.

Countries around the world have defended themselves against SARS-CoV-2 using travel restrictions, national lockdowns, facemask policies, and other non-pharmaceutical interventions to stop the spread of SARS-CoV-2, and evaluating these population-level actions requires different tools than what we present in this paper (i.e., there is no path to an RCT for some public health measures). Here, we use the tools of pharmacoepidemiology, a field spanning clinical pharmacology and epidemiology, to study the effects of drugs in large numbers of people in order to estimate probabilities of beneficial and/or adverse effects. We introduce this body of knowledge as 10 rules for retrospective pharmacoepidemiological analyses designed to evaluate a treatment hypothesis (see Figure 1 for the 10 rules and Table 1 for common vocabulary). These rules are the result of a community effort, including academic, health care, nonprofit, and industry contributors, to establish a set of best practices for retrospective analyses. A retrospective analysis aims to estimate the comparative effectiveness of one treatment vs. another (e.g., a new treatment vs. the standard care) using real-world evidence (Office of the Commissioner, 2020) obtained from preexisting data such as electronic health records (EHR), insurance claims databases, or health care registries. We embark on a retrospective analysis knowing that it should not stand alone as the sole evidence supporting adoption of a new treatment; observational study evidence should be considered *suggestive* rather than *conclusive*. A retrospective analysis can contribute a body of real-world evidence as a supplement to the medical theory supporting the treatment and any preclinical studies conducted *in vitro* and/or *in vivo*, all of which combine to inform decisions about whether and how to pursue a randomized trial.

**TABLE 1:** This table of common terms provides working definitions for vocabulary appearing in the following 10 rules.

Term	Definition
causal effect	a difference between two potential outcomes, one where the individual is exposed and one where the individual is unexposed (or exposed to a different treatment)
cohort	a group of people with some defining characteristic (e.g., a disease)
comorbidity	a co-occurring medical condition in addition to the primary condition
comparison group/control group	groups that identify individuals who have not received the treatment of interest and have instead received either no treatment or a different treatment; often denoted as unexposed
confounders	variables satisfying three properties: they are associated with the outcome (i.e., risk factors), they are associated with the exposure (i.e., they are unequally distributed among the exposure groups), and they are not effects of the exposure
confounding	a bias in the measure of a treatment effect resulting from treatments and outcomes sharing a common cause
confounding by indication	when the condition or indication prompting exposure also affects the outcome (e.g., if the exposure of interest in a drug-repurposing study is a diabetes drug, individuals with prior prescriptions for this drug likely have diabetes and might be expected to have worse outcomes)
directed acyclic graph (DAG)	a tool for depicting assumptions and selecting variables to include in the analysis using directed arrows representing cause-effect relationships
exposure	the treatment or experience that defines the intervention under investigation (e.g., takes a drug, undergoes physical therapy, etc.)
external validity	how generalizable the finding is beyond the study population
internal validity	the degree to which the observed result is believed to be attributable to the observed treatment and not unseen factors
outcome	a clearly defined, measurable indicator of health status (e.g., blood pressure level, disease recurrence within a specified timeline, or in-hospital death)
pharmacoepidemiology	a field spanning clinical pharmacology and epidemiology focused on studying the effects of drugs in large numbers of people in order to estimate probabilities of beneficial and/or adverse effects
potential outcomes	what an individual would have counterfactually experienced when either exposed or not exposed (e.g., received a drug vs. no drug)
preregistration	registering the details of a study -- hypotheses, methods, analysis plans -- before it is conducted
retrospective analysis	an estimation of the comparative effectiveness of one treatment vs. another (e.g., a new treatment vs. the standard care) using real-world evidence obtained from preexisting data such as electronic health records (EHR), insurance claims databases, or health care registries
selection bias	a distortion of the treatment-outcome association principally resulting from the lack of randomized treatment assignment
sensitivity analysis	analyses conducted to observe the study result's sensitivity to a change in population/definition/method/assumption
surrogate outcomes	synthetic or permuted outcomes used to blind investigators to the real study results until various code and definition validations are complete
trial emulation	designing an observational study to mimic a randomized controlled trial with similar goals

## COVID-19 Study

Here we introduce a potential COVID-19 pharmaceutical treatment to discuss the 10 rules more concretely. Prior work indicates that certain alpha-1 adrenergic receptor antagonists (alpha blockers) disrupt cytokine storm syndromes, a pathological hyperinflammatory response associated with respiratory infection and other diseases (Staedtke et al., 2018; Koenecke et al., 2021; Thomsen et al., 2021). Subsequently, others determined that hyperinflammation is implicated in morbidity and mortality in COVID-19 patients (Mehta et al., 2020; Li et al., 2021). Many COVID-19 patients were already taking alpha blockers prior to infection for unrelated, chronic medical conditions. Consistent use of doxazosin (a particular alpha blocker) prior to COVID-19 diagnosis is the exposure of interest, and the goal is to estimate its effectiveness for preventing in-hospital death.

We are now ready to dig into the 10 rules. Rules 1–3 describe three guiding principles for a retrospective pharmacoepidemiological analysis. Rules 4–7 discuss key preparations for the analysis. Rules 8–9 address how to develop and refine the analysis plan. Rule 10 concludes with executing, summarizing, and reporting the results to facilitate replicating and extending them. Each rule could have its own paper or book chapter (and in many cases they do), and we expand the discussion of each rule considerably in the supplementary material to explain the concrete, actionable steps the rules require.

## Guiding Principles: Build and Focus the Team

### Rule 1: Form a Multidisciplinary Team

Get the right people involved at the start, in the middle, and at the end. Every step of the way you are going to need to make decisions about the medical rationale for the proposed exposure, treatment practices in clinics and hospitals, the nuances of relevant data stores and common coding practices, the study design, and the statistical analyses and interpretation of results. Specifically, high-quality retrospective analyses depend on input from committed individuals with different domain expertise: medical, data sources, epidemiology, and causal analysis.

#### COVID-19 Study

Clinicians provide insights into the differences between exposed (those prescribed doxazosin) and unexposed groups; understanding the conditions that lead to treatment is critical in designing the study. Clinical experience working with patients diagnosed with COVID-19 is also helpful for gaining insight into the dynamics of COVID-19 testing and patient care. For example, the protocols for testing and admitting patients have varied over place and time, especially early in the crisis. In an evolving pandemic, these factors motivate accounting for changing patient populations; failing to do so could result in biased estimates of treatment effects.

A COVID-19 study presents unique challenges. First, there is an urgency to rapidly (and comprehensively) assess a proposed exposure. Second, the landscape changes while the study is underway: new datasets emerge and published results change attitudes for different treatments. Third, near-constant sharing of ideas and work products is crucial, but the study team members are likely isolated. Getting feedback early and often from all parties is crucial for reducing time-to-iterate without sacrificing research quality (London and Kimmelman, 2020). While still ensuring HIPAA protections are appropriately observed, tools like Slack, GitHub, and Google Docs for conversing, collaborating on code, and writing, respectively, facilitate the kind of rapid progress that is otherwise hard to achieve.

### Rule 2: Emulate a Randomized Controlled Trial

Design your observational study to mimic — as closely as possible — a randomized controlled trial with similar goals, an approach known as *trial emulation* (Rubin, 2004; Rosenbaum, 2010; Hernán and Robins, 2016; Dickerman et al., 2019). Carefully consider what you measure, when you measure it, and in whom you measure it. Draw a CONSORT diagram of the ideal RCT you wish you could run (Begg et al., 1996). Emulating an RCT should ideally include preregistration of the study and analysis plans (described in Rule 9).

#### COVID-19 Study

Our retrospective analysis should emulate the desired RCT investigating doxazosin as a prophylactic treatment for severe symptoms among patients with COVID-19 (Konig et al., 2020). The trial would target older adults, a group who appears to have the greatest risk of adverse outcomes from COVID-19 (D-19 Provisional Coun, 2020). Emulating this trial requires focusing on the same patient group in our retrospective analysis. Without random exposure assignment, the retrospective study must identify people taking doxazosin prior to a COVID-19 diagnosis. In the United States, many older adults take doxazosin for conditions including hypertension and benign prostatic hyperplasia (BPH). Thus, emulating a trial in older adults would be both meaningful (by studying the impact on a group at risk for adverse outcomes from COVID-19) and feasible (since observing doxazosin use in this group is likely). There is a cost, however, to targeting a subset of the population; the study can lose external validity for other patient groups (Holdcroft, 2007).

### Rule 3: Realize That Rule 2 Is Impossible and Proceed Carefully

In an observational study, our choices of what to measure and in whom to measure it are limited by what data already exists. Even more concerning, our inability to randomize exposure assignment introduces categories of variables that we worry less about in randomized controlled trials, most notably confounders. Confounders satisfy three properties: they are associated with the outcome (i.e., risk factors), they are associated with the exposure (i.e., they are unequally distributed among the exposure groups), and they are not effects of the exposure (Jager et al., 2008). If not observed and sufficiently addressed, confounders lead to confounding, which is a bias in the measure of a treatment effect resulting from treatments and outcomes sharing a common cause (Hernán and Robins, 2020). Review the different kinds of covariates that can exist in a causal analysis of observational data and how each can impact causal estimates (see Rule 5). Confounding by indication is likely to occur in observational data, and the primary concern in your observational study is the identification and mitigation of potential confounders. Your analysis will therefore need to address confoundedness as evidenced by observed differences in the covariate distributions of the various exposure groups, and you can conduct descriptive analysis characterizing observed differences between treatment and control groups to complement qualitative information gathering about the treatment assignment process in order to guide your thinking about what variables will be necessary to include in the data to mitigate confounding.

#### COVID-19 Study

Expanding on our previous observation that older people are more likely to be taking doxazosin, we now consider how confounding can emerge in an observational study and the importance of addressing it. Without the deliberate recruitment and randomization of an RCT, doxazosin use will be concentrated among the older individuals eligible for our study because both hypertension and BPH prevalence increase with age (Partin et al., 1991; AlGhatrif et al., 2013). COVID-19 outcomes appear to be worse with increased age, suggesting that age is a confounder we must address. Even if doxazosin is effective at reducing all-cause mortality, doxazosin is disproportionately prescribed to older people who disproportionately have worse outcomes. Unless we account for age, a truly beneficial treatment effect could be estimated with negative bias (possibly making the treatment appear harmful). This example from our COVID-19 observational study highlights the reasoning required to identify important covariates to consider in our analyses.

## Preparation Phase: Establish the Hypotheses and Acquire Resources to Evaluate Them

### Rule 4: Formalize the Research Goal

Specify the exposure in terms of quantity, duration, frequency, and recency. Define the comparison groups of interest (e.g., define *unexposed*). Bias (e.g., *selection bias*) can arise from many sources in an observational study, but it fundamentally stems from the lack of randomized exposure assignment, resulting in the construction of a control group having different concerns than the treated group with regard to censoring, missing data, self-selection, or even eligibility for treatment (Hernán et al., 2004). While confounding by indication is almost guaranteed to be present in non-experimental pharmacoepidemiology research and will be addressed in other rules, we highlight the importance now of identifying comparison groups in which every individual theoretically has some probability of receiving the proposed treatment. An example of questionable comparison group construction could be comparing two groups with the same disease but where the two groups take different drugs based on significant differences in disease severity (e.g., metformin for less advanced type 2 diabetes mellitus vs. insulin for more advanced type 2 diabetes mellitus). Next, define an outcome that is specific, measurable, and sufficient to answer the research question. Finally, formalize your hypotheses (i.e., specify the null and alternative, sidedness, primary vs. secondary exposures and outcomes).

#### COVID-19 Study

A pharmaceutical study considers a particular drug, dosage, recency, and duration by using prescription records to qualify a patient as either exposed or unexposed to the medication under investigation (e.g., doxazosin, ≥4 mg daily, prescription valid through COVID-19 diagnosis date, continuous use reflected by total days’ supply covering 80% of the previous 3 months — a quantity known as the medication possession ratio or MPR (Andrade et al., 2006)). When quantifying duration and recency, multiple filled prescriptions for a drug better indicate continued use than a single fill that may have gone unused. Prescriptions lasting until some key date (possibly allowing for skipped doses) provide better evidence that the drug was in use on the date of interest. Unfortunately, researchers are usually unable to confirm the medication was consumed as intended. Some patients deviate from the prescribed drug regimen, and this is often unobservable; we therefore conduct *intent-to-treat* analysis by grouping patients according to inferred exposures revealed in prescription records (Gupta, 2011). The comparison group might include anyone who does not meet the exposure definition, only people who have not taken the proposed drug for a specified length of time, or perhaps only people who have never taken any alpha blocker. Importantly, the comparison group should not be made up of people who cannot take alpha blockers for reasons that could relate to their health outcomes.

As COVID-19 was entering its first peak, many countries’ chief concerns were ventilator resources and anticipated deaths. Outcomes related to ventilator dependence or mortality may be of particular interest. We found that using ventilator dependence as an outcome is often problematic for two reasons. First, ventilator usage depends on the standard of care with respect to administering ventilator resources at a particular time and place, and the severity of patients in the data as well as treatment protocols differed substantially by time and place during the pandemic. Second, insufficient ventilator availability and inconsistent ventilator coding practices makes ventilator dependence a complicated outcome in some places. All-cause mortality is not completely unaffected by the changing practices related to ventilators, but mortality proves to be the more clearly defined outcome of ultimate importance. Since we cannot quantify the exact role of COVID-19 in hospital deaths, the best practice is to use all-cause mortality as the primary outcome of interest.

### Rule 5: Identify and Reason About Potential Confounders

**FIGURE 2:**
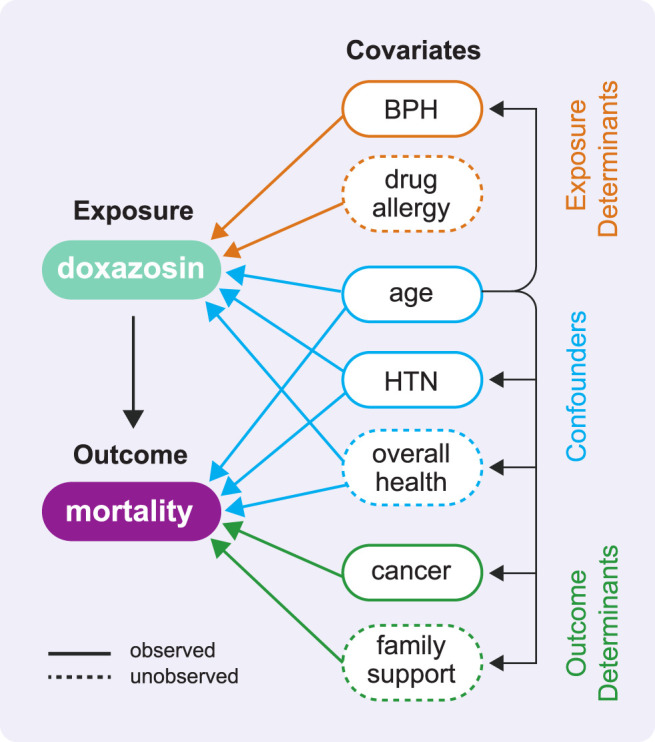
This directed acyclic graph (DAG) shows the types of variable relationships described in Rule 3 using the example COVID-19 study. A DAG has no cycles, which means no variable can cause itself, either directly or through one or more other variables. In our effort to estimate the causal effect of doxazosin on mortality, this DAG helps us identify which variables will be important to adjust for in our analyses (in reality, this diagram would include many more variables of these same types). It is the set of confounders that has the ability to distort the association between exposure and outcome as revealed by the arrows leading from each confounder to both the exposure and the outcome. We highlight two observed confounders: the demographic confounder age and the comorbidity confounder hypertension (HTN). We also depict the unobserved confounder overall health, which we might attempt to measure using indicators of overall health like frequency and duration of recent inpatient stays.

Confounders will be present; make every effort to observe these confounders and adjust for them appropriately. Include standard demographic variables, relevant comorbidities, and a comorbidity index and/or other indicators of overall health. Note that identifying confounders before you have data will help you better assess the utility of candidate datasets. Organize your understanding of the key variables with a causal diagram (see Figure 2). A directed acyclic graph (DAG) is a powerful way to depict the causal relationships in your analysis (Greenland et al., 1999; Pearl, 2009) and examine potential biases your analysis might permit (VanderWeele et al., 2008). Bias might result from an unobserved confounder that is not measured in the data and therefore cannot be adjusted for in the analysis; a significant unobserved confounder can invalidate all results obtained from the study. Thinking through each variable and the corresponding existence and direction of arrows (representing both observed and unobserved cause-effect relationships) helps prevent unknowingly inviting bias into your analysis and mitigate potential sources of bias that you do include. Following procedures for identifying a minimally sufficient adjustment set (MSAS) of confounders in a DAG (VanderWeele et al., 2008) can eliminate adjustment-induced bias. Ultimately, a DAG provides an excellent visual representation of the known or assumed relationships between variables and helps identify the necessary variables to adjust for to minimize confounding in a multivariable analysis. Know that no matter what you do, you will likely still have unobserved confounding (we describe sensitivity analyses to quantify the magnitude of this issue in the Rule 9 supplement).

#### COVID-19 Study

Several alpha blockers (doxazosin included) have an FDA indication for hypertension, so we expect the exposed population will have higher rates of hypertension, a condition that might lead to worse outcomes. Relevant comorbidities that serve as confounders per clinicians’ expertise include sex, age, diabetes mellitus, hypertension, cardiovascular disease, and chronic obstructive pulmonary disease. For the doxazosin hypothesis, patient location has significance as prescription practices and the standard of care for relevant conditions vary around the world. Even with these considerations, unobserved confounding can still affect a study’s results. Unobserved confounding is one reason why the results of observational studies of hydroxychloroquine have differed from those of RCTs (Hernandez et al., 2020).

### Rule 6: Operationalize the Target Population

Select the target population for your observational study to reflect the intended RCT population. Refine the potential study population by setting the inclusion and exclusion criteria to minimize confounding. Consider the impact of refining the target population on both internal validity (focused on groups the study includes) and external validity (focused on groups to which the findings might extend).

#### COVID-19 Study

In a COVID-19 retrospective cohort study, the defining characteristic of patients in the cohort is a COVID-19 diagnosis. In our observational study, the exposure was administered prior to the COVID-19 diagnosis. Using a post-treatment variable to define the cohort can introduce post-treatment bias, so choosing to select the sample on the basis of a post-treatment variable (COVID-19 diagnosis) implies we believe the exposure has no impact on one’s susceptibility to infection and likelihood of diagnosis. We are aware of no evidence that taking doxazosin changes one’s susceptibility to SARS-CoV-2 infection; doxazosin could, however, affect whether a person is diagnosed by mitigating symptoms to a degree that a patient self-treats rather than seeing a doctor to receive a formal diagnosis. Early in the pandemic, COVID-19 tests were only available in inpatient environments and were reserved for the sickest patients. Individuals were urged to stay home until they truly needed hospital resources. This led to many unobserved, undiagnosed patients. We cannot estimate the treatment effect in this population as we do not observe the qualifying condition: a COVID-19 diagnosis. Later in the pandemic, we face the same problem, but for a different reason; widespread community testing facilitates diagnoses, but these test results and diagnoses may not enter a patient’s health records or claims history (both common data sources for retrospective studies). We could again lose visibility of milder cases where a patient recovers at home, limiting our assessment to the severe cases warranting hospitalization. This is a notable limitation of defining the cohort by a COVID-19 diagnosis.

We focus the doxazosin study on older patients because this group is at high risk of adverse outcomes from COVID-19. Older men in the United States take doxazosin at a far higher rate than women, primarily because doxazosin is a treatment for BPH. Compared to other men of the same age, a prior BPH diagnosis is not expected to have any impact on COVID-19 outcomes. We now make the consequential restriction to focus the study on older men, allowing us to capture many exposed individuals with no above-average risk for negative outcomes. This target patient population attempts to minimize the impact of unobserved confounding. While this may be appealing, the exclusions have important implications. Pragmatically, reducing the population under consideration may reduce statistical power by limiting the sample size. Societally, focusing the study exclusively on older men limits the study’s internal validity to older men. It will take additional assumptions and/or further analyses to extend the study’s findings to women and young people.

### Rule 7: Get the Best Data for the Study

Invest time in getting access to the best possible data for your study such that your desired study definitions can be realized. Know what your data source contains, where it originated, and how it was assembled. Know the biases and limitations of candidate datasets. Identify the target population using carefully selected, standardized diagnosis and/or procedure codes. Identify chronic comorbidities using standard condition code sets (Chronic Conditions Data Warehouse, 2020) and sufficient patient histories.

#### COVID-19 Study

Identifying COVID-19 patients can be difficult because of the nonexistence of COVID-19-specific International Classification of Diseases (ICD) codes early on in the pandemic. It was only on April 1, 2020 that ICD-10 U07.1 was introduced for a confirmed diagnosis of COVID-19, and adoption of this code for billing purposes remained variable and inconsistent for some time. Using an established, community-derived definition for the COVID-19 population is recommended (e.g., as provided by the National COVID Cohort Collaborative - N3C (National COVID Cohort Collaborative, 2020)). COVID-19 population definitions often divide into two groups: *COVID-narrow* includes confirmed COVID-19 diagnoses while *COVID-broad* adds suspected COVID-19 patients who have not been tested but exhibit multiple COVID-19 symptoms. Large hospitals that treated thousands of COVID-19 patients and performed in-house testing (e.g., Mount Sinai Hospital in New York City) are best situated to precisely construct a COVID-19 cohort (Wang et al., 2020).

In the early stages of a pandemic, finding a well-curated, sufficiently sized data set to test your hypothesis on the novel disease may be impossible. Expert clinical input may identify a suitable substitute for COVID-19 that reflects the same symptoms and disease progression your treatment is theorized to target (e.g., cytokine storm syndrome resulting from acute respiratory distress or pneumonia). Identifying such a disease with established coding and extensive patient records can jumpstart your research while the data practices surrounding an emerging pandemic stabilize.

The hypothetical doxazosin study requires access to each individual’s inpatient, outpatient, and prescription drug history for at least the year leading up to COVID-19 diagnosis. Clinical data from the U.S. Veterans Health Administration (VHA) is an ideal candidate data set for this type of study for several reasons. Older adults are well represented in the VA health care system, typically with extensive patient histories. This reduces the likelihood of having the insufficient patient histories that sometimes accompany individuals in a claims database who have recently changed employers. In addition, the VA health system would have comprehensive records: diagnoses, procedures, prescription drug use, doctors’ notes, in-hospital medications received, and lab results.

## Analysis Planning Phase: Develop and Refine the Analysis Plan

### Rule 8: Explore and Model Your Data With Surrogate Outcomes

Use permuted outcomes or synthetic data (Koenecke and Varian, 2020) as you build and test your analysis code to prevent being influenced by any premature results. First, examine the univariate and pairwise distributions of the covariates that will be used in the analysis. Second, examine all covariate distributions after stratification by exposure group and/or time period, compute each individual’s propensity for treatment (i.e., estimate a propensity score), and obtain better empirical overlap using propensity trimming (Lee et al., 2011). A propensity score reflects the probability that an individual would receive treatment (i.e., belong to the exposed group) on the basis of observed covariates. To counter confounding by indication, a variety of analytical techniques employ propensity scores to balance the exposed and unexposed groups by matching or weighting using propensity scores, which assign greater weight to the unexposed individuals who appear more similar to the exposed individuals in terms of the observed covariates. Third, begin modeling with an unadjusted modeling approach (e.g., simple logistic regression) to establish a baseline treatment effect estimate. Finally, use additional modeling approaches that adjust for confounders (e.g., doubly robust methods (Bang and Robins, 2005) employing propensity scores and covariate adjustment in the outcome models), favoring methods that seek covariate balance.

#### COVID-19 Study

Examining the covariate distributions of the exposed and unexposed groups will likely reveal that doxazosin users are generally older and have more comorbidities than non-users. Unadjusted models with no consideration of age would likely compare a younger, healthier unexposed group to an older, less healthy exposed group. We addressed this problem by including age as an observed confounder and by establishing inclusion/exclusion criteria that ensured anyone in the study could reasonably have been exposed to doxazosin. Now, we further exclude observations exhibiting extremely high or low propensity for treatment (on the basis of all covariates, not just age); this could include the extremely young, old, healthy, sick, etc. Extreme propensities indicate that almost all similar units share the same treatment assignment, such that there is limited information in the data about how similar individuals would have fared if their treatment assignment had been different.

### Rule 9: Augment the Main Analysis With Extensive Sensitivity Analyses

Plan a thorough assessment of the robustness of your results to the many choices made along the way to estimating a treatment effect. Start by conducting supplementary analysis designed to illustrate clearly the role of observed confounders for both treatment assignment and outcome modeling, as this can build intuition about what factors are likely important in these processes (Athey et al., 2017). Quantify the extent of unobserved confounding required to change your conclusions (Rosenbaum and Rubin, 1983; Rosenbaum, 2010; VanderWeele and Ding, 2017) (i.e., determine how correlated an unobserved variable must be with the exposure and outcome to nullify any perceived treatment effect). Assess the robustness of your results to different modeling techniques, hyperparameters, outcome definitions, exposure definitions, inclusion/exclusion criteria, and other aspects of the study design. Explore additional sets of covariates, including different comorbidities and indicators of temporal health trends. Conduct negative outcome experiments and treatment control experiments (Lipsitch et al., 2010). Refine, lock in, and preregister your formal analysis plan before examining any real model outputs using the true outcome data.

#### COVID-19 Study

Robustness checks for a doxazosin study assess the impact of making adjustments to the treatment, outcome, and population definitions. We can test our hypothesis on both a COVID-narrow cohort and a COVID-broad cohort. Our confidence in the treatment will also be tied to how well our results hold up to changing the medication possession ratio and changing the post-diagnosis window we are monitoring for all-cause mortality. We can explore additional covariates beyond chronic comorbidities that may indicate increased health concerns closer to the COVID-19 diagnosis (e.g., other inpatient stays within 2 months of diagnosis).

## Execution Phase: Execute the Analysis Plan and Report The Results

### Rule 10: Execute, Summarize, and Share (With Caveats)

Execute your analysis plan with the true outcome data once you are satisfied with the quality of your data set and have sufficiently tested your code. If necessary, make the smallest possible refinements to your analysis plan and execute again, always ensuring you report deviations from your preregistered plan. Give your reader something that looks like what they are used to seeing (i.e., conventional measures of treatment effect, standard tables and figures). Explicitly describe the limitations of your study. Provide all the necessary method descriptions and code to facilitate replication.

#### COVID-19 Study

We include a CONSORT diagram to show the split of doxazosin users and nonusers in the dataset, followed by their respective outcome counts, to help visualize the study like an RCT. We are targeting a clinical research-savvy audience including clinical trialists, so we present the treatment effect as an odds ratio (OR), which is a familiar metric for the likely readers. We define our null hypothesis as OR = 1 (i.e., the exposure does not change the odds of the outcome occurring). We then assess doxazosin to be beneficial if we find OR < 1. We present the associated confidence interval (CI) to convey the precision of our treatment effect estimate. Together, the OR and CI indicate the strength of evidence supporting further investigation of the doxazosin hypothesis.

## Conclusion

As the pandemic is far from over, especially in lower resource countries and communities, we see the value both now and in future pandemics of responsibly investigating the efficacy of inexpensive, repurposed drugs as early treatment options while we wait for vaccine development, mass production, and global distribution. The primary benefits associated with conducting these investigations with retrospective analyses lie in reducing costs and increasing speed relative to running an RCT (assuming the RCT would be feasible and ethical). Moreover, retrospective pharmacoepidemiological analyses can be run even when no patients are available (e.g., after everyone is vaccinated) to learn more about potential treatments for future pandemics. Retrospective analyses make it easier to explore a variety of treatments with limited time and other resources, setting the stage for an RCT to test the most promising interventions. In the COVID-19 era, these are valuable benefits, but they come with a cost. The challenges facing retrospective analyses arise from the requirement to use data generated without a particular study in mind. Unlike an RCT, where researchers are able to decide exactly who will be recruited to participate, which exposure(s) will be assessed (e.g., drug, dosage, frequency, duration, etc.), and which outcome(s) will be measured, the observational study approach described here limits the researcher to only those definitions of exposure, outcome, confounders, and sample population that can be realized with available data. This places a significant burden on the researcher to determine whether the desired retrospective analysis is possible to conduct with available data. When the time and cost savings of performing a study with observational data outweigh the costs of constrained data collection and study design, using these 10 rules as a guide will support the execution of a rigorous retrospective pharmacoepidemiological analysis that speeds the time to clinical trials and, hopefully, proven effective treatments for patients.

## Supplement: How to Follow These 10 Rules

This supplement serves to explain in detail the many recommendations made in the 10 rule paragraphs in the main text. Individual sentences in the rule paragraphs generally correspond to one or more paragraphs in this supplement explaining why the recommendation was made and how to satisfy its requirements.

### Guiding Principles: Build and Focus the Team

#### Rule 1 Supplement: Form a Multidisciplinary Team

The main text states we require continuous input reflecting different kinds of domain expertise: medical, data sources, epidemiology, and causal analysis. Medical expertise ensures the study remains medically coherent while decisions are made throughout the design of the study. Data source expertise (including medical terminologists) can expedite the process of finding, accessing, and understanding relevant data sources and corresponding coding conventions, while also making known their potential limitations. The expertise in epidemiology that comes from working with observational health data ensures the study design and study definitions meet accepted standards in the literature (e.g., defining treatments, conditions, and other health indicators with observational data). Causal inference expertise ensures the use of appropriate analysis methods to support making a causal claim. The degree to which each expert contributes in each successive rule varies, but it is difficult to underestimate the value of assembling this group at the start.

#### Rule 2 Supplement: Emulate a Randomized Controlled Trial

Design your observational study to mimic — as closely as possible — a randomized controlled trial with similar goals, an approach known as *trial emulation* (Rubin, 2004; Rosenbaum, 2010; Hernán and Robins, 2016; Dickerman et al., 2019). To start down this path, we must first clearly state the research objective. Most likely the clinician(s) on the team will be the source of the medical hypothesis. What is the pathophysiological mechanism this study seeks to understand? Which exposure(s) might reasonably affect this mechanism? Which subset of the population do we think the exposure(s) will benefit? Who could reasonably be eligible to receive the proposed exposure? Which measurable outcome(s) will reveal the efficacy of the proposed exposure(s)? Which analyses will be needed to do the appropriate comparisons? These details will continue to be refined as we think through the remaining rules, and we will rely on the team’s clinical expertise to ensure any refinements continue to support the primary research objective.

Carefully consider what you measure, when you measure it, and in whom you measure it. It can be helpful to lay out key aspects of the study design just as would be done in an RCT using a CONSORT flow diagram (Begg et al., 1996) and other observational study reporting standards (Benchimol et al., 2015; Langan et al., 2018). For example, a person considered for trial participation must be deemed eligible for the trial at the time of exposure group assignment, which must then occur before any follow-up periods begin or outcomes are observed. Suppose your ideal trial has an exclusion criterion barring participation of anyone with a history of heart problems. Heart problems that surface at some point after a person receives the exposure might be visible in observational data; since post-exposure health problems could not have been observed for the purposes of RCT enrollment, we ignore them when deciding the eligibility of patients for observational studies (Dickerman et al., 2019).

Preregister your study and analysis plan just like an RCT. Before an RCT begins, the individuals running the trial will have already amassed a corpus of information about the relationship between the exposure and outcome (e.g., in preclinical data). They have used this information to design the trial and get approval from an institutional review board (IRB). Given this information, the study plan is fixed prior to collecting any patient information in the actual trial phase. The trial emulation proposed in this paper similarly promotes an exploratory data analysis and modeling phase that uses surrogate outcome data to refine the analysis plan before committing to a final outcome analysis to be run on actual outcome data (discussed further in Rules 8–10). Preregistering the study and documenting a final analysis plan avoids several pitfalls associated with the recent replication crisis: questionable research practices (John et al., 2012), HARKing -- hypothesizing after results are known (Kerr, 1998), gardens of forking paths (Gelman and Loken, 2014), and p-hacking (Schuemie et al., 2018). Avoiding these pitfalls is particularly important in a pandemic study since even preliminary results from individual studies can have profound policy and public health implications, as well as implications for ongoing clinical trials (Piller and Travis, 2020). While the idea of preregistration in observational studies continues to grow in popularity, the effectiveness of the practice has notable limitations. For example, often the data has already been collected and been available for research prior to a study’s preregistration, making it hard to verify whether preregistration actually preceded the reported analysis.

Recall the assumptions necessary in order to make a causal claim. A key premise of an RCT is that the exposure assignment is random; in particular, exposure assignment is independent of factors that affect patient outcomes. To facilitate random exposure assignment, the study inclusion/exclusion criteria in an RCT must be designed to ensure that every trial participant can reasonably be assigned to any exposure group. Random exposure in an RCT is then accomplished by arbitrarily assigning people to either of the exposed or unexposed groups using a coin flip, or in the case of a stratified RCT, a coin flip that depends only on observed pretreatment factors. Our inability to achieve random exposure in an observational study means we must make some assumptions to estimate treatment effects when we do not observe all of the patients’ potential outcomes (e.g., both the exposed outcome and the unexposed outcome for each patient when there are two exposure groups). Here we state one of the acceptable sets of assumptions for conducting a retrospective analysis. First, theoretical *overlap* ensures that for any possible set of values of pretreatment traits (i.e., patient characteristics), there is a non-zero probability of being in either group. Lack of overlap might occur in practice if patients with certain characteristics are either excluded from the exposure group or always assigned to the exposure group (e.g., the exposed group only contains adults while the unexposed group contains both children and adults). Second, the property of *unconfoundedness* (also known as *strong ignorability*) ensures that exposure assignment is independent of the potential outcomes given the observed covariates. Of these assumptions, overlap can be verified empirically, but there is no test to prove we have satisfied the unconfoundedness assumption.

Finally, we assume (both in observational studies and RCTs) that the specific exposure assigned to one individual does not interfere with the exposure or potential outcomes of any other individual in the study. For example, interference may occur when one patient in an RCT receives the exposure and is cured, which may then free up hospital resources to the benefit of an unexposed patient in an adjacent room. Furthermore, the exposure must be the same for everyone in an exposure group (e.g., identical drug regimen). Together, these two criteria comprise the Stable Unit Treatment Value Assumption (SUTVA) (Imbens and Rubin, 2015).

A gold-standard randomized controlled trial satisfies all of these assumptions by construction; however, the lack of randomized exposure assignments in an observational study means there is significant work associated with emulating an RCT as closely as possible. It is almost certain that meaningful differences exist between the exposed and unexposed groups, and that the factors that differ are also related to outcomes. Confounding by indication is likely to occur in observational data, and the primary concern in your observational study is the identification and mitigation of potential confounders, which is the basis of Rule 3.

#### Rule 3 Supplement: Realize That Rule 2 Is Impossible and Proceed Carefully

Recall the different kinds of covariates in a causal analysis and how each can impact causal estimates. The lack of randomized exposure assignment in an observational study forces us to address the pretreatment variables that we observe in our data. Given that we are seeking to determine the causal effect of an exposure on an outcome, there are three types of observed variables that can exist in relation to this study. The first, outcome determinants, affect the outcome but do not directly affect the exposure. While you can include outcome determinants in your analysis to improve the precision of your causal effect estimate, a causal analysis can proceed without them. The second, exposure determinants, affect the exposure but do not directly affect the outcome. Exposure determinants will also not affect our analysis because there will be zero covariance between the outcome and the exposure conditional on these variables. A note beyond the scope of this paper: econometric analysis can reveal whether any of these exposure determinants is a *strong instrumental variable.* In this case, a separate instrumental variables analysis (Hernán and Robins, 2006) is preferable for studying the effect of the exposure on the outcome by exploiting the fact that the instrumental variable’s effect on the outcome definitionally only exists via the exposure. The third type of variable affects both the exposure and the outcome; these are known as *confounders* and are the essential variables to identify for your study.

Think hard (and then think harder) about confounders for your study. As defined in the main text, confounders satisfy three properties: they are associated with the outcome (i.e., risk factors), they are associated with the exposure (i.e., they are unequally distributed among the exposure groups), and they are not effects of the exposure (Jager et al., 2008). Identifying important confounders requires collaborating with specialists who can make appropriate clinical recommendations; for example, one might learn that there exists a *comorbidity* (an additional, simultaneously occurring disease or condition) for which patients would be taking the exposure drug. This comorbidity would be considered the indication or reason for prescribing the drug (as listed in the US prescribing information, though clinicians may prescribe for other reasons). Perhaps this comorbidity typically leads to worse outcomes given the worse overall health of these patients. Such a comorbidity would be a confounder; other common confounders include demographic variables such as age and sex.

Make a plan to address non-overlap and confoundedness. First, we must recognize that we only have data for observed confounders (as opposed to unobserved confounders, for which we have no data, and which in general lead to bias in estimates of causal effects). To address non-overlap, we must ensure that for any observed combination of confounder values, there are patients with very similar observed combinations of confounder values in each of the exposed and unexposed groups, even if presence in one group is more likely than another. If there are any combinations of confounder values for which the probability of exposure is either zero or one, it is impossible to estimate the treatment effect for patients with those confounder values. As a practical matter, the associated observations should be excluded to achieve overlap; the target population for which we estimate the treatment effect is correspondingly narrowed. To deal with confounders, we must mitigate the non-random exposure assignment in our data by ensuring similar distributions of confounder values between exposed and unexposed groups. There are two main approaches to doing so: outcome modeling and covariate balancing; when combined, the approaches may be doubly robust in that they are still valid if errors are made in either modeling or balancing (but not both), as discussed in more detail in Rule 8. Outcome modeling builds a model of the relationship between covariates and outcomes, allowing the analyst to adjust for the impact of differences in covariates across groups on differences in outcomes. Covariate balancing attempts to reweight or subsample from data such that the exposed and unexposed groups are comparable in terms of covariates, so that the covariates are no longer associated with exposure in the new, reweighted data; this can be accomplished, for example, through sample restriction with inclusion/exclusion criteria, reweighting by inverse propensity scores (probability of assignment), stratification, or matching (Stuart, 2010) on confounders. Note that almost certainly there exists unobserved confounding in any observational study, and unobserved confounding distorts our view of the exposure-outcome relationship. If we believe there is an important unobserved confounder, it may be appropriate to abandon the study or use a different approach (e.g., instrumental variables analysis). We will address unobserved confounding in greater detail in Rule 5 and how to account for it with sensitivity analyses in Rule 9.

### Preparation Phase: Establish the Hypotheses and Acquire Resources to Evaluate Them

#### Rule 4 Supplement: Formalize the Research Goal

Specify the exposure in terms of quantity, duration, frequency, and recency. The study’s purpose is to evaluate the efficacy of this exposure, and this should dictate your first step in formalizing the research goal. The proposed exposure in a pharmaceutical-based hypothesis involves identifying a set of drugs for testing. At a minimum, this requires labeling each patient in the study as exposed or unexposed to one of the drugs in question; doing so requires completing two tasks. The first task is for the clinician team to specify the precise list of drugs and corresponding dosages they wish to include as the exposure drug set based on the pathophysiological mechanism they wish to target. The second task is to determine the timing of the observed drug exposure. For example, does it matter if the patient is a current, recent, or historical user of the drug at the time of the patient’s diagnosis (Pazzagli et al., 2018)? How long must a patient have used the drug to be part of the exposed group? These questions directly relate to the pathophysiological mechanism the proposed treatment aims to target, and the answers to these questions may have implications for the degree to which the study can truly emulate an RCT. Note that every consideration above also applies to analysis of a non-pharmaceutical exposure. Investigating the effectiveness of a non-pharmaceutical therapy requires the same attention be given to defining the precise list of qualifying therapies as well as the quantity, duration, frequency, and recency of any treatment a patient received.

Define the comparison groups of interest (e.g., define unexposed). If you could do a randomized experiment, what other exposure groups would you randomly assign people to for comparison? In a pharmaceutical study, this could include taking a placebo, taking an active comparator (an alternative treatment known to be effective), or even taking the same drug according to a different regimen. Defining a comparison condition requires the same level of detail required for the exposure definitions. Most likely the comparison condition represents the existing standard of care, and the purpose of the study is to see if the hypothesized exposure provides an improvement over the standard care. As you define the exposure and comparison conditions, it may well be the case that some individuals meet none of these group definitions and must accordingly be excluded from the study. For example, some patients may fall just short of qualifying as exposed (e.g., too few days on the proposed drug treatment, too small a dosage), but their classification as unexposed would be inappropriate as well.

Define an outcome that is specific, measurable, and sufficient to answer the research question. Defining an outcome includes clearly stating exactly what will be measured, when it will be measured, and how it will be measured for all patients in the study. The outcome must be observable in a consistent manner for all patients in your study. Thoughtful consideration should be given to the followup time required to observe the outcome in both exposed and unexposed patients. Additionally, for outcomes other than mortality, competing risks may prevent observing the outcome of interest (e.g., loss to follow-up in a lengthy study).

Formalize your hypotheses. At this point in the team’s preparation for the study we have clearly defined the exposure(s) and outcome(s) and are ready to articulate the causal effect of interest. This involves clearly stating the specific null and alternative hypotheses your analysis will test; determine if a one-sided or two-sided test is more appropriate for your medical hypothesis. Commit to the primary and secondary exposure and outcome definitions, target population, and outcome-focused results you believe will produce a credible analysis. Note that the hypothesis is based on definitions that reflect what you hope to observe, and they may not be what you can actually find in an available data set (discussed further in Rule 7).

##### Example Application of Rule 4 to the COVID-19 Study

This retrospective study estimates the causal effect of baseline use of doxazosin (daily dose ≥4 mg with prescriptions covering the day of COVID-19 diagnosis and at least 80% of the previous 3 months) compared to nonuse (no prescriptions for any alpha blocker in the previous year) on reducing all-cause mortality in adults over 45 years old who have been diagnosed with COVID-19. We state the following hypotheses for the odds ratio (OR) associated with the treatment effect on all-cause mortality:

$$H_{0}:OR\geq 1,\;H_{A}:OR<1.$$

#### Rule 5 Supplement: Identify and Reason About Potential Confounders

Confounders will be present; make every effort to observe these confounders and adjust for them appropriately. Consider a study wherein patients are prescribed a drug to treat a certain disease with varying degrees of severity. A high dosage tends to be prescribed for patients with a more severe case of the disease, whereas a low dosage tends to be prescribed for patients with a less severe case of the disease. It would be no surprise to find that patients with severe cases have worse outcomes as a group - even if the drug (and dosage) they are taking is the best option for their individual situations. In observational data, dosage level is inherently related to severity of illness. Hence, severity of illness is a confounder because it affects the exposure-outcome relationship; if left unobserved, severity of illness could irreparably confound any study results. The circumstances surrounding the administration of an exposure can also make observing confounders challenging. For example, suppose we are studying the efficacy of a drug for preventing death from an acute condition, and the drug is typically given as a last resort to patients who are nearing death from that condition. Then it may be difficult or impossible to observe the factors that affect both exposure and outcome, since not all factors that lead a physician to believe that the patient is at high risk of death will be recorded. During some time periods in the COVID-19 pandemic, different drugs (such as hydroxychloroquine) were given off-label to the sickest patients. In such circumstances, receiving the drug is an indication that the patient was very ill. In contrast, if we study exposure to a drug that was prescribed for a chronic condition long before a patient developed COVID-19, then exposure will not be determined by the patient’s severity of symptoms from COVID-19. For example, some underlying factor such as hypertension might be related to both drug exposure and risk of poor outcomes from COVID-19, so it will still be important to carefully adjust for all such factors.

Include standard demographic variables. Common demographic covariates such as sex and age (including nonlinear transformations like age-squared) are standard confounders to consider, appearing in nearly all epidemiological models. Another variable to consider is the time or location of the sample-defining diagnosis (e.g., a positive lab test or clinician diagnosis). Diseases like influenza often change from year to year in terms of which strains are more prevalent, and the geography of outbreaks may not be uniform. Depending on how fast a disease mutates or the standard of care changes, capturing the year, month, or even week of diagnosis, and/or hospital or patient location, may be important covariates when examining observed outcomes.

Include relevant comorbidities. A confounding comorbidity is one that impacts both exposure assignment and outcomes. Other comorbidities may be unrelated to the proposed exposure but could still be helpful as proxies for confounders by identifying which patients are already at higher risk for severe outcomes based on components of their health beyond basic demographics (e.g., cancer or heart failure). Still other comorbidities might serve as proxies for the proposed treatment; running an analysis that includes these comorbidities may lead to “post-treatment bias” because the comorbidities would appear as concurrent treatments, hence reducing the estimated treatment effect of the actual treatment. Post-treatment bias can also result from considering post-treatment traits. For example, controlling for emphysema when examining the causal effect of smoking on lung cancer would likely transfer some of the treatment effect from smoking to emphysema, which we might assume to have resulted from smoking. Choosing to consider a confounder that was observed post-treatment requires a deliberate assessment of the potential causal relationship between the exposure and the observed trait. For example, if an observed comorbidity is of a chronic nature, it may be unlikely that a recent exposure caused the comorbidity; most likely the unrelated condition prompting the exposure led to the healthcare encounter where the comorbidity was first diagnosed. Another class of variable to avoid is known as a collider. A collider is a variable that can be considered an effect of both the exposure and the outcome; controlling for such a variable introduces bias in the effect estimate.

Include a comorbidity index and/or other indicators of overall health. The Elixhauser comorbidity score (Elixhauser et al., 1998) and Charlson comorbidity index (D’Hoore et al., 1993) are two established measures combining various observed medical conditions in order to serve as more general indicators of overall health than an individual, disease-indicating covariate. The potential for unobserved, general health problems can also be addressed by looking at a patient’s recent health care encounters and prescription data. Encounter-related covariates may include the number of inpatient or outpatient visits occurring in the year preceding the relevant diagnosis, the duration of inpatient stays (i.e., the number of days the patient had been in the hospital in the previous year), and indicators for whether the comorbidities listed above were observed closer in time to the relevant diagnosis (e.g., within two months prior rather than within one year prior). Considering the recency of documented health concerns is useful for establishing whether a declining health trend exists both at the individual level and at the level of comparing different exposure groups. You may also want to consider certain procedures in addition to diagnoses (e.g., colonoscopies, flu shots (Jackson et al., 2006)), which can also serve as indicators of overall health and/or access to health care. As with all of our confounders, remember to ensure that any indicators of overall health only capture pretreatment health conditions.

Know that no matter what you do, you will likely still have unobserved confounding. Failing to include unobserved confounders in an analysis leads to omitted variable bias, which violates the unconfoundedness assumption. As indicated above, the missing confounders we are most concerned with relate to unobserved indications of poor or declining health; however, these may not always be available. If you determine a set of critical confounding variables and find that some are unobservable (either directly or via a proxy variable), we can investigate the potential magnitude of this unconfoundedness violation (in some cases, your proposed study may be too flawed to justify pursuing it). There is certainly a bit of tension here as we perform analysis under the assumption of unconfoundedness while simultaneously acknowledging the likelihood of unobserved confounding. We address this tension with sensitivity analyses described in Rule 9.

##### Example Application of Rule 5 to the COVID-19 Study

This retrospective study considers the following confounders: sex, age, diabetes mellitus, hypertension, cardiovascular disease (acute myocardial infarction, ischemic heart disease, heart failure), chronic obstructive pulmonary disease, patient location, Elixhauser comorbidity score, inpatient stays in the prior year, inpatient stays in the prior 2 months, inpatient days in the prior year, and inpatient days in the prior 2 months.

#### Rule 6 Supplement: Operationalize the Target Population

Select the target population for your observational study to reflect the intended RCT population. Patient selection is a key task in RCTs, and an observational study emulating an RCT should implement the same inclusion and exclusion criteria as the RCT. Given that an RCT likely excludes individuals with certain comorbidities, one benefit of an observational study is the opportunity to conduct a subanalysis of individuals that the RCT would exclude.

Refine the potential study population by expanding the inclusion and exclusion criteria to minimize confounding. In Rule 5 we described many types of potential confounders; in Rule 6 our objective is to find a subset of the population who may receive the exposure of interest for reasons that have minimal expected impact on the outcome of interest (i.e., minimal confounding); importantly, these individuals should also include candidates to remain unexposed. There is no rule of thumb for this, but rather it is through the creative efforts of your team that you can specify a target population refinement that can still potentially answer the research question while significantly reducing confounding. Note that changing the sample inherently changes the estimand, and there is often a tradeoff between studying the population that is of greatest interest and studying the population where estimates are most credible.

Consider the impact of refining the target population on internal and external validity. Minimizing confounding is desirable as it increases the internal validity of the study, but excluding certain groups from the study may limit the external validity of the results to only the refined population under study (Imai et al., 2008; Rudolph et al., 2014). Consider again a scenario where a drug is administered in some cases for conditions with serious health risks and in other cases as more of a lifestyle drug. If we exclude from our study any patients with the more serious condition, we can likely achieve more similar exposed and unexposed groups, which is important for attributing any difference in expected outcome to the exposure under investigation. The cost is not knowing how those with the more serious condition fare with the exposure versus without the exposure. Additionally, there is an important emerging literature on demographic fairness with regard to clinical studies (Holdcroft, 2007). Be careful in your efforts to minimize confounding so that you do not unintentionally or unnecessarily exclude a portion of the population that also requires study.

##### Example Applications of Rule 6 to the COVID-19 Study

1) This retrospective study focuses on adults over 45 years old to maintain internal validity for all older adults. 2) This retrospective study focuses on adult men over 45 years old to minimize confounding by focusing on a large group of people that use doxazosin for a condition unlikely to affect COVID-19 outcomes (BPH).

#### Rule 7 Supplement: Get the Best Data for the Study

Invest time in getting access to the best possible data for your study. Above all else, this means the target patient population is sufficiently represented in the dataset. Recognize that data access and sharing may be challenging; any health care data you use will often have data access restrictions due to legal and/or privacy concerns, proprietary interests, or other competitive barriers (Byrd et al., 2020). Typically, IRB approval, an IRB waiver for de-identified data, or business associate agreements enable data access and permit its use for your specific research objective.

Know what your data source contains, where it originated, and how it was assembled. Having someone on the team who knows the data source well helps the team avoid the early stumbles that inevitably happen while working with new data. The best data sources will capture data on the population, exposure, outcomes, and covariates relevant for a study. Once you acquire access to potential datasets, consider the reliability of the data collection (e.g., provenance, missingness, measurement error, trends over time, and sampling or representativeness of the target population). While we recommend defining your ideal exposure(s), outcome(s), and target population first, you may have to revise some of these definitions to be compatible with the existing dataset or combination of data sources (e.g., claims data, labs, or electronic health records from multiple participating hospitals).

Know the biases and limitations of candidate datasets. It is likely the case that no single data source is sufficient to represent the broader population. The ideal data source would have extensive electronic health records with thorough patient histories documenting inpatient and outpatient encounters, diagnosed conditions, and drug prescription and fill data. Outside of national healthcare systems or other integrated systems such as the US Veterans Health Administration (VHA) and Kaiser Permanente, obtaining all relevant information about a specific patient from a single source is rare. Often, hospital data will not have extensive pre-hospitalization data (if any), and claims databases will lack the rich details of hospital records (e.g., clinicians’ notes and lab results). Further, observed outcomes in patient groups from different data sources may not always be indicative of what is expected in the broader population. Certain types of hospitals (e.g., tertiary care centers) may handle more advanced cases of a disease and have higher rates of certain outcomes in their electronic health records data. Some insurance claims databases may only represent the portion of the population that is employed, has healthcare insurance, and has demonstrated access to healthcare services. Each data source may also be idiosyncratic according to varying standards of care and coding practices for the time, location, and patient groups it represents. The information that appears in health data can also reflect payment systems and incentives; for example, minor hospital procedures may not appear in claims databases because insurers may not pay for them directly. It is important to know and understand these issues before trying to run your models across different datasets, only to be confused by the inconsistent results. The best approach is to evaluate your hypothesis using as many appropriate data sources as possible and look for consistently observed effects across data sets.

Obtain a sample of the target population using carefully selected, standardized codes. The typical way of identifying patients for a cohort study involves selecting patients with a documented record of a particular disease or medical procedure, most often by means of an International Classification of Diseases (ICD) code (e.g., ICD-10-CM Clinical Modification). Many diseases and procedures have a large number of codes delineating the various subtypes of the disease (e.g., pneumonia) or procedure (e.g., mechanical ventilation), so a careful inspection of the potential list of qualifying condition codes is necessary to properly define the intended sample. If possible, attempt to validate the cohort by also checking for confirmatory lab tests and/or prescribed medications, which may or may not be available in your data.

Identify chronic comorbidities using standard condition code sets and sufficient patient histories. The data you will need for a cohort study must contain some mechanism for observing the confounders you identified in Rule 5. Diagnoses for comorbidities, much like the diagnoses used to define our target patient population, can include a broad range of ICD codes for each disease or condition. Identify comorbidities by using a standard set of ICD codes that medical researchers generally agree encompass the common comorbid conditions, such as the Chronic Conditions Data Warehouse (CCW) (Chronic Conditions Data Warehouse, 2020) produced by the Centers for Medicare & Medicaid Services (CMS). You will need reasonably long-duration patient histories (e.g., 12+ months of inpatient and outpatient records preceding the diagnosis meriting inclusion in your study's cohort) to ensure adequate opportunity to observe relevant comorbidities in patient records. As a general rule for most chronic conditions, we recommend considering a patient to be positive for a given chronic condition if any of the listed condition codes in a standard code set is referenced as a diagnosis on any inpatient or outpatient record in the 12 months preceding the qualifying diagnosis. In turn, researchers should exclude any patient that cannot be tracked in the data for that entire lookback period (e.g., in insurance claims data, if the patient was not continuously enrolled during that time). The clinicians and data source experts on the team should determine whether any alternate criteria should be considered (e.g., multiple codes, multiple occurrences, different lookback period, lab values, and procedure codes).

Make your study definitions realizable in your data. It should be expected in database-facilitated research that not all desired quantities may be available. For example, rarely can we know what medication a person actually consumed; instead, we observe what was prescribed and filled. An insurance claims database does not generally record indicators of a patient’s lifestyle such as body mass index (BMI), alcohol use, and smoking status (though they could be very useful); they may not record certain demographic and socioeconomic data (also relevant for many diseases and hypotheses). Instead, an insurance company needs to know which diagnoses were given and which procedures were administered for claims reimbursement purposes. As you look for data that allow you to operationalize your study definitions for exposure, outcome, confounders, and target population, you may be forced to adjust those definitions to reflect what is in the data. You must carefully assess whether what you do observe is close enough to what you wish you could observe to be sufficient for the research question.

##### Example Application of Rule 7 to the COVID-19 Study

This retrospective study uses Veterans Health Administration data with patients identified according to the National COVID Cohort Collaborative’s COVID-broad criteria. Pretreatment comorbidities are identified by searching each patient’s inpatient and outpatient records (electronic health records or insurance claims) for the presence of a qualifying ICD code for each of several comorbid conditions according to the comorbidity-specific ICD code sets provided by the Chronic Conditions Data Warehouse.

### Analysis Planning Phase: Develop and Refine the Analysis Plan

#### Rule 8 Supplement: Explore and Model Your Data With Surrogate Outcomes

Use permuted outcomes or synthetic data as you build and test your analysis code. In an RCT, blinding prevents patients and clinicians from knowing exposure group assignments, which might affect their respective actions. In observational studies, the concept of blinding relates to only seeing what you have to see to accomplish a certain task. Research team members can be blinded to the exposure, the outcome, and potentially even the hypothesis (Berman and Parker, 2016). We start this rule by blinding ourselves to the outcome because all code goes through a debugging phase, and there is a risk that, at least subconsciously, you might be influenced by frequently seeing a range of results from different methods, confounder/covariate sets, etc. As you proceed with your analysis, you may discover that certain covariates are either sufficiently sparse or so highly correlated with other covariates that issues of numerical stability arise with certain modeling approaches. As you encounter these issues and fine-tune your list of covariates, it is best that these modifications be made without subjective bias arising from prematurely observing any effect estimates. Remember, the purpose here is to specify the details of the analysis plan and to implement working code, not to produce a final causal effect estimate just yet. If a step can be performed with surrogate outcome data for the purpose of testing, it should be.

Examine the univariate and pairwise distributions of the variables (or covariates) that will be used in the analysis. This serves to assess any issues with missingness, data entry errors, and the accuracy of any constructed variables. Also important is the opportunity to assess these distributions for their adherence to known or believed attributes of the population under study.

Examine all covariate distributions after stratification by exposure group and/or time period. A key claim in any retrospective analysis, as mentioned in Rule 3, is that the exposed and unexposed groups either have similar covariate distributions or that the authors have done something to address the fact that the distributions are meaningfully different. The difference in the exposed and unexposed groups’ covariate distributions is typically referred to as “covariate balance,” which should be calculated and visualized before and after employing certain types of models (Austin, 2009).

Achieve better empirical overlap using propensity trimming. Propensity scores quantify each patient’s likelihood of receiving the exposure conditional on the observed covariates. There may exist observations in your data that possess combinations of covariate values that are only ever observed in either the exposed group or the unexposed group, but not in both (leading to uncommonly high or low propensity scores). This violates the overlap assumption we required in Rule 2 (while this statement applies as written to categorical variables, a relaxed version still applies to continuous variables where exact matches are unlikely). A standard technique to maintain overlap is to remove such observations from the data by trimming on the basis of propensity scores (i.e., restricting the sample to areas with propensity score overlap). There are many common approaches to calculating propensity scores; the R packages *grf*, *twang*, and *MatchIt* calculate propensity scores using honest forests, generalized boosted models, and logistic regression, respectively (Ho et al., 2011; Athey et al., 2019; Ridgeway et al., 2020) (note that some machine learning models are characterized by bias or inconsistency in estimates of propensity scores, and so properties such as honesty as implemented in *grf* may be important if machine learning methods are used in propensity score estimation). The distributions of propensity scores in the exposed and unexposed groups are then used to identify and trim (remove) observations that are in the extremes of these distributions and have few or no counterparts in the other exposure group with a similar propensity score. This process ensures that in every region of the preserved covariate distribution, there exist observations in both the exposed and unexposed groups. Thus, overlap ensures we are estimating a causal effect over regions of the covariate distribution supported by data rather than through extrapolation. Achieving this overlap is how we most closely emulate the RCT reality in which every patient has some positive probability of assignment to each exposure group. Note that the groups as a whole could still look quite different (e.g., in terms of comorbidity prevalence).

Use an unadjusted modeling approach to establish a baseline treatment effect estimate. Assuming two exposure groups and two potential outcomes, start with any method operating on 2-by-2 contingency tables; you could use Fisher’s exact test, the chi-square test for association, or a basic logistic regression model to evaluate the exposure-outcome association with no adjustment for any confounders. Importantly, you want to obtain point estimates and confidence intervals (CI) from these methods as we are concerned with the magnitude and precision of the treatment effect estimate. Despite our repeated emphasis on identifying and accounting for confounders, having an unadjusted model result that is compatible with the adjusted model results (described next) demonstrates that you have not reached your final treatment effect estimate simply by selecting a favorable set of covariates. When unadjusted and adjusted results disagree, one explanation could be dissimilarities in the covariate distributions of the exposed and unexposed groups. For example, if certain ages or comorbidities are not approximately equally represented in all exposure groups, controlling for such covariates could potentially change the sign of the estimated treatment effect. This could be evidence that your inclusion/exclusion criteria do not by themselves go far enough to yield similar exposed and unexposed groups.

Adjust for confounders, favoring methods that both adjust for outcomes and seek covariate balance. Methods that adjust for outcomes build a model mapping covariates to expected outcomes and then adjust for these differences when estimating treatment effects. Ordinary least squares or logistic regression are common methods for outcome adjustment; machine learning methods can also be used, but caution must be exercised, as there is a danger that regularization might omit or insufficiently adjust for confounders, creating bias (Belloni et al., 2014). Covariate balance goes beyond ensuring overlap: now the exposed and unexposed groups must resemble each other in their covariate distributions. More simply, observed values in the exposed group should occur with similar frequency in the unexposed group (either by weighting or excluding observations). Methods that accomplish this include inverse propensity-weighted (IPW) average of outcomes and matching (Rubin, 2001; Stuart, 2010; Jackson et al., 2017).

There are many choices of regression methods that adjust for confounders; among these are a set of methods known as doubly robust methods. A doubly robust estimator is one that employs both a propensity score model and an outcome regression model in such a way that if either model is correctly specified, the resulting causal effect estimator is statistically consistent (Bang and Robins, 2005). An example of a doubly robust method is inverse propensity-weighted (IPW) regression. Inverse propensity score weighting seeks covariate balance by weighting unexposed observations in the regression according to the inverse of their propensity scores (Austin and Stuart, 2015). Thus, observations that do not resemble exposed observations contribute less to the treatment effect estimate, and unexposed observations resembling exposed observations count more. This type of weighting has the effect of attempting to achieve covariate balance by weighting observations rather than excluding observations. Other examples of doubly robust methods include augmented inverse propensity weighting or AIPW regression and causal forests (Bang and Robins, 2005; Athey et al., 2019). We note that if machine learning techniques are used to estimate outcome models and propensity scores in AIPW methods, it is important to use cross-fitting, where the outcome adjustment and propensity score model for a given observation is estimated excluding that observation. When out-of-bag estimates are used with random forest methods, this will happen automatically, but with other methods, the analyst must estimate multiple versions of these models on different folds of the data.

As an alternative to the above doubly robust methods, one can employ matching methods to stratify the sample into one group per exposed observation. Groups or “matched pairs” are sized such that each exposed observation has a corresponding number of unexposed observations according to a specified match ratio. Importantly, the matching process should only retain the exposed observations for which an acceptable number of unexposed observations serve as good matches. This is the nearest you can get to seeing how a person’s potential outcomes might be different on the basis of exposure. Matching can be accomplished many ways, including on the basis of propensity score or Mahalanobis distance (Stuart, 2010). To estimate the causal effect of the exposure on the outcome in the matched pairs, one might use the Cochran-Mantel-Haenszel test (Mantel and Haenszel, 1959) to evaluate the collective evidence presented by a series of 2 × 2 contingency tables documenting the exposure-outcome counts in each matched pair. The process of matching could produce a potentially much smaller data set that attempts to achieve covariate balance by excluding observations.

For methods that rely on covariate balance as part of the approach to adjust for confounders, it is critical to conduct appropriate diagnostics to see if these approaches achieved acceptable covariate balance. If you are unable to achieve reasonable covariate balance between exposed and unexposed individuals, you have likely discovered fundamental differences in the two groups that no modeling approach can reliably overcome (Glynn, 2017).

##### Example Application of Rule 8 to the COVID-19 Study

We first create a permuted copy of the outcome variable representing in-hospital death. We use the R package *grf* to estimate propensity scores (i.e., real exposure assignments as a function of the pretreatment traits identified in Rule 5). We then trim the sample to retain the overlapping region of the exposed and unexposed propensity score distributions by keeping scores above the maximum of the two distributions’ first percentiles and below the minimum of the two distributions’ 99th percentiles. With the remaining sample, we perform an unadjusted analysis of the exposure-outcome relationship with Fisher’s exact test (OR, CI, and *p*-value obtained with base R Fisher exact test). We conduct an adjusted analysis using the same pretreatment traits in an inverse propensity-weighted (IPW) logistic regression (OR, CI, and *p*-value obtained with the R package *survey*). We use the R package *MatchIt* to execute 5:1 Mahalanobis distance-based matching (identify five unique, unexposed matches for each exposed patient) on the same pretreatment traits (OR, CI, and *p*-value obtained with base R Cochran-Mantel-Haenszel test). Finally, we assess the covariate balance achieved by IPW and matching by calculating and visualizing standardized differences of means for included covariates. Executing all of these steps with permuted outcomes helps us debug code, identify potential incompatibilities with our data and selected methods, and conduct meaningful diagnostics for covariate balancing methods — all with zero awareness of the impact on our treatment effect estimates.

#### Rule 9 Supplement: Augment the Main Analysis With Extensive Sensitivity Analyses

Plan a thorough assessment of the robustness of your results to the various choices you made on the way to calculating an estimated treatment effect. Maybe you left something out that could explain everything (i.e., an unobserved confounder). Do alternative design and analysis approaches yield similar results? A secondary set of analyses could include adjusting for covariates with nonlinearities or time lags; you could also try different regression or propensity estimation methods. There could be many reasonable specifications for your model; to avoid tying your results to a set of arbitrary decisions, one way to evaluate a collection of reasonable models is to observe the distribution of resulting effect estimates using specification curve analysis (Simonsohn et al., 2019). Exploring different exposure or outcome definitions, covariates, designs, and analysis techniques also helps measure the sensitivity of your results to the specific choices you made along the way. Assessing robustness is by itself a comprehensive analysis.

Quantify the extent of unobserved confounding required to change your conclusions. If you are using observational health data to perform your study, you should expect that unobserved confounding exists; the difficulty lies in estimating how serious it is. There is no test for unobserved confounding (neither its existence nor its impact, given that it is unobserved), yet it likely exists in nearly all observational studies. This reality is what makes having domain experts carefully reason through confounder specification so critical. Starting with (Rosenbaum and Rubin, 1983), numerous approaches have been proposed that generally aim to estimate how strongly correlated an unobserved confounder would have to be to either the exposure, the outcome, or both, to move the estimated treatment effect to the null (Rosenbaum, 2010). Then you can reason about how likely it is that such a confounder might exist and is either unknown or unmeasurable. One such method for assessing unobserved confounding is the E-value (VanderWeele and Ding, 2017).

Assess the robustness of your results to choices regarding specific modeling techniques, hyperparameters, etc. One way to accomplish this involves trying a range of estimation approaches. Compare the treatment effect estimates from a range of doubly robust methods, for example. Use a variety of machine learning methods to estimate propensity scores and outcome models in doubly robust methods such as AIPW, or use approaches such as residual balancing (Athey et al., 2018) that do not rely on having an easy-to-estimate propensity model. The reason to augment your analysis by testing multiple approaches is to see if the obtained results were sensitive to the specific methods you chose to employ. While the methods introduced so far are designed to estimate average treatment effects for a population or some subset of the population, knowing whether the treatment effect is generally constant across the considered group can be very important. To explore this, one can construct causal trees to estimate heterogeneous treatment effects or HTE (Athey and Imbens, 2016).

Assess the robustness of your results to modifications in the study definitions and study design. You can make small changes to the definitions of the exposed and unexposed groups as well as the outcomes and confounders. For example, to identify a patient as a user of a particular drug, adjust the aforementioned medication possession ratio or look-back period in the exposure definition (i.e., ensuring a medication supply of more than 50, 70, or 90% of days within a look-back period of 90, 180, or 365 days). You can consider different recency requirements such as whether the most recent prescription spanned the inpatient admission date of interest. For an outcome like all-cause mortality, you could explore all-cause mortality in the hospital or within 7, 14, 30, or 60 days of diagnosis. Comorbidity identification could employ different code sets and/or a different look-back period. You may also consider adjusting for additional (or only a subset of) potential confounders within your models, to observe the extent to which confounder choice matters. The objective here is to see whether or not any observed treatment effect is simply a chance result stemming from a very specific set of definitions. Some of these changes are sufficient to change the study design. For example, defining the unexposed group to only include users of a different, comparable drug is known as the *active comparator design*, which can be an effective approach for minimizing confounding as the exposed and unexposed groups will be more similar (Yoshida et al., 2015). If we define the exposed group to only include new users of a drug, thus ensuring observed comorbidities existed before exposure and eliminating concerns over prevalent user bias, we are implementing a new user or incident user design. There are many study designs to choose from (e.g., prevalent user, incident user, active comparator, etc.), and each design deserves thoughtful consideration regarding the implications it has for the study in question and physiological mechanism under investigation. While investigating robustness to changes in study design can provide more evidence for the hypothesis, it can also help identify potential sources of unobserved confounding when different designs lead to different conclusions.

Explore additional sets of covariates, including different comorbidities and indicators of temporal health trends. Covariate sufficiency is the notion that no other covariate can meaningfully supplement what we have learned from the already identified covariates (Stone, 1993; VanderWeele and Shpitser, 2013). We can explore the sufficiency of our identified confounders by observing how results are impacted by the inclusion of other comorbidities. We can also explore the impact of differing time trends in the health of the exposed and unexposed populations. If one exposure group was observed to be getting sicker faster in the months before the target inpatient admission, that could warrant different expectations for outcomes in the exposed and unexposed groups. Your confounder definitions may have difficulty addressing not only the presence of a condition, but also its recency and its severity. Many comorbidities have their own severity indices (e.g., Diabetes Complications Severity Index), but viewing all the data required to compute these scores may not always be possible in certain data sets (e.g., claims data lacks lab results). Observing health decline is thus challenging; consider examining recent inpatient stays and other medical encounters as signs of declining health that may not otherwise be captured in existing confounder definitions.

Conduct negative outcome experiments and treatment control experiments. In a negative outcome experiment (Lipsitch et al., 2010), your goal is to assess whether the hypothesized exposure has an apparent benefit that extends to an outcome it could not reasonably impact (i.e., no medical theory connecting the exposure to the outcome). A negative outcome experiment is run to study the effect of the proposed treatment on an outcome not associated with that treatment. Here, we should expect to find no favorable treatment effect; otherwise, there is likely unobserved confounding contributing to better outcomes for the exposed group. A treatment control experiment is run to study a different treatment with no known connection to the outcome of interest; you should observe no protective effect of this different treatment on your original outcome. Again, if you see a benefit where there should be no benefit, the logical conclusion is the presence of unobserved confounding.

Refine, lock in, and preregister your formal analysis plan before examining any real model outputs using the true outcome data. Preregistration for observational studies involves uploading a detailed analysis plan to a study registry like the ones supported by the US National Library of Medicine (*clinicaltrials.gov*) and the Center for Open Science (*cos.io/initiatives/prereg*). While we encourage preregistration, in some cases it may not be possible to preregister an analysis plan before ever seeing the data; your understanding of the data prior to working with it may be too limited to make preregistration worthwhile. Preregistering your analysis plan is an attempt at transparency regarding what is exploratory and what is confirmatory in your final analysis. You may discover some things while exploring your data and testing your proposed statistical methods that require you to refine prior decisions. Maybe your set of confounders and outcome determinants is incompatible with a method you’ve chosen because one variable is too rarely observed or is too highly correlated with another variable. This is fine; you can make the necessary changes to your analysis plan with no fear of p-hacking because you were not using real outcomes (due to outcome permutation or synthetic data generation per Rule 8) and have not seen an effect estimate yet. Your preregistered analysis plan may include a range of exposures, outcomes, and modeling approaches you intend to evaluate, but you must clearly articulate from among these which combination you commit to reporting as your primary result. Define your primary result with a clear statement of the hypothesis, details of the modeling approach, and definitions for the cohort, treatment, outcome, and confounders.

##### Example Application of Rule 9 to the COVID-19 Study

We assess robustness to unobserved confounding with the E-value. We estimate the treatment effect with different exposure definitions, specifically combining 50, 70, and 90% MPR with 90-, 180-, and 365-days exposure windows. We estimate the treatment effect using AIPW and heterogeneous treatment effect with causal trees as supplementary methods. We consider mortality within 30 days of diagnosis as an alternative to in-hospital mortality. We perform a negative treatment control experiment with triptans as the exposure. We perform negative outcome control experiments using accidental injuries and non-prostate cancer as alternate outcomes.

### Execution Phase: Execute the Analysis Plan and Report the Results

#### Rule 10 Supplement: Execute, Summarize, and Share (With Caveats)

Execute your analysis plan with the true outcome data once you are satisfied with the quality of your data set and have sufficiently tested your code. A significant responsibility of your team at this point is to stick to the proposed analysis plan. Other outcomes and exposures may appear to have a stronger effect than what is observed for the primary outcome and exposure, but there was significant thought and clinical expertise applied to these decisions in the planning phase of the study. There is danger in evaluating a host of different outcomes and only reporting the most favorable outcome(s); this greatly increases the potential for a Type I error, meaning that you could be reporting a treatment effect that does not actually exist.

If necessary, make the smallest possible refinements to your analysis plan and execute again. Even with all your planning, there is a chance that your analysis plan cannot be executed as-is. For example, you may discover that a rarely observed confounder in your data is perfectly predictive of the outcome in one of your exposure groups. This perfect separation of the data could cause your preferred method to fail, leaving you no choice but to change one of your selected methods or your selected confounders or both. If this happens, all is not lost. Simply make the minimal possible change necessary to conduct your analysis, and then note in your publication how you had to amend your analysis plan and what potential impacts your change may have had on your results.

Give your reader something that looks like what they are used to seeing. If your retrospective analysis has the stated purpose of motivating a clinical trial, write your results like a clinical trial paper. Include a CONSORT flow diagram to help the reader visualize important properties of your sample. Understand how the intended audience expects to see results reported for the selected outcomes. The clinician audience you are writing for is accustomed to seeing odds ratios with corresponding confidence intervals to describe treatment effects. Presenting results in a conventional way eliminates one potential obstacle your audience may face when evaluating your work. While much attention is given to your primary result, your results in total are more than just an OR and a confidence interval; report the results of your sensitivity analyses as well to convey the robustness of your finding.

Explicitly include in your reporting the limitations of your study. You have not just completed an RCT; instead, you performed an observational study modeled after an RCT, but with many limitations and assumptions. Your biggest enemy is unobserved confounding, and it might be the case that it has seriously affected your results; however, if done well, your retrospective analysis may be just what is needed to generate the momentum and funding required to evaluate your idea in a clinical trial (Vandenbroucke, 2004). Alternatively, your analysis may actually provide evidence against the hypothesized exposure. Reporting negative results is just as important; your work can help ensure limited resources are spent on more promising treatments.

Provide all the necessary details to facilitate replication. You took great care in constructing and executing a comprehensive analysis plan; as you prepare to disseminate your findings, sharing those details matters. More than just your results, some readers will want to know everything necessary to reproduce your analysis. This means you should expect to provide details about the data used, including source and provenance as well as the codes (e.g., ICD) used to define the target patient population, inclusion/exclusion criteria, the exposure(s), the outcome(s), and any confounders. It can’t be assumed that a reader will be able to guess your definitions without having them explicitly written out. Other researchers could sensibly reach many different definitions of what they believe you meant by the various outcomes, exposures, and confounders listed in your retrospective analysis. Providing text definitions, formulas, and ICD-code lookup tables ensures that any other attempts to implement your definitions are able to accurately do so. Providing all of this information in the standard organization of a clinical trial paper will help your clinical audience find the key pieces of information they need to be able to envision the trial you are emulating.

Facilitate replication by providing analysis code. You may also want to create an open-source software package (e.g., R/Python) for dynamic exploration of a data set and/or to facilitate replication of your analysis on other data sets. It is likely the case that other entities (e.g., a hospital, an insurance company, or a country) cannot legally share their data set with you; you likely have the same restrictions preventing sharing your data outside your own institution. To get around these restrictions and make replication as easy as possible, you can share instructions and code for building the data set and running your desired analysis. Whether you provide a well-documented collection of scripts in an online Git repository or a more formal software package, if you want to see replication of your results (e.g., to support an RCT you aim to start), you have an incentive to provide a reusable codebase that can facilitate rapid replication of results in other data sets as well as provide a means of quickly exploring alternate hypotheses.

### Future Directions

These 10 rules are intended as introductory guidelines to one small piece of the complicated world of observational studies; there is much more to learn and consider than is offered here. Perhaps most importantly, we acknowledge this paper’s role in summarizing a framework for retrospective pharmacoepidemiological analyses, not as a template for all types of retrospective studies (e.g., investigating lockdowns and facemask policy effectiveness against the spread of COVID-19). Several other ideas came up in the course of establishing these 10 rules that fell just short of earning their own rules. Some are not yet standard practice but are growing in popularity, and others are even more aspirational. Among these are notions of sample splitting (Fafchamps and Labonne, 2017) and model pooling. Sample splitting in the world of machine learning is standard practice, but typically the machine learning problem is one of prediction where there exists validation data, making it possible to know how correct a model’s predictions are and therefore tune the model. The causal inference framework differs on both those counts: prediction is not the goal, and there exists no validation data to help us see if we have missed any unobserved confounders. While sample splitting may not always be necessary, when doubly robust techniques are used and machine learning methods are used to estimate outcome models or propensity scores, cross-fitting is needed to apply existing theory (Chernozhukov et al., 2018; Athey et al., 2019); we recommend that approach as discussed in Rule 8. There is still interest, however, in using synthetic data generation techniques such as generative adversarial networks (Beaulieu-Jones Brett et al., 2019; Athey et al., 2021) and standard training/test splits for routine tasks like evaluating a constructed feature definition and validating code. Employing these or related techniques aims to facilitate completion of necessary tasks without being influenced by real-world results. Another growing area of interest is in the pooling of data and models from observational studies (Bareinboim and Pearl, 2016). Privacy concerns often restrict the pooling of data, but these concerns do not apply to the pooling of models. Pooling different linear models is nothing new, but combining nonlinear models shows promise for providing doubly robust causal estimates with lower variance, even when the source models have different covariates as inputs. As more research on these and other areas continues, it is likely we will see the associated advances make their way into some of the key ideas we have captured here.
